# Parent autonomy support and undergraduates’ academic engagement in online learning: the mediate role of self-regulation

**DOI:** 10.1186/s41155-024-00330-1

**Published:** 2024-11-18

**Authors:** Lili Song, Qiqi Zhan, LuSheng Cao, Runfeng Luo

**Affiliations:** 1 Xiamen City University/Xiamen Open University, Xiamen, China; 2 Institute of Education, Xiamen University, Xiamen, China; 3 School of Teacher Education, Shaoxing University, Yuecheng District, No. 900 Chengnan Avenue, Shaoxing City, Zhejiang Province 312000 People’s Republic of China; 4 JuYuan Primary School, Taizhou, China

**Keywords:** Online learning, Parent autonomy support, Self-regulation, Behavioral engagement, Emotional engagement, Cognitive engagement

## Abstract

**Background:**

The role of parent support for adolescents has been validated in online learning. However, less attention has been paid to undergraduates.

**Main body:**

The research used self-reported questionnaires to investigate the mediating effects of self-regulation in parent autonomy support and academic engagement (cognitive, behavioral, and emotional dimensions) within the online environment in the context of Chinese culture.

**Objective:**

The present study recruited 1908 undergraduates in China.

**Methods:**

The students completed measures of parent autonomy support, self-regulation and three sub-dimensions of academic engagement.

**Results:**

The results indicated that parent autonomy support exerted a direct and significant effect on the three sub-dimensions of academic engagement in online learning. Self-regulation partially mediated the relations between parent autonomy support and three sub-dimensions of academic engagement.

**Conclusion:**

These findings showed parents should autonomously support students to promote their cognitive, behavioral, and emotional engagement. Moreover, the partial mediation explained how parent autonomy support affected three sub-dimensions of academic engagement. Limitations and educational implications were also discussed.

**Supplementary Information:**

The online version contains supplementary material available at 10.1186/s41155-024-00330-1.

## Introduction

During the COVID-19 pandemic, a nationwide lockdown was mandated in China. The enforced closure of schools and universities led to a significant shift in student learning and the rapid popularity of online classes (Zhoc et al., 2022). When studying online, undergraduates experienced feelings of isolation from peers and professors (Kaufmann & Vallade, 2020), exhibited diminished academic motivation (Sindiani et al., 2020), and an elevated likelihood of dropping out (Xiao, Liu, Shang, and Huang, 2021). Consequently, it is worth exploring how to enhance the effectiveness of online learning to increase accessibility, elevate the quality of online education, and better prepare for “lifelong” learning opportunities (Appana, 2008).

Academic engagement in online learning serves as a crucial indicator for effectively assessing the quality of online education (Gao et al., 2022). Academic engagement, encompassing cognitive, behavioral, and emotional dimensions, reflects the extent of a student’s commitment and effort in the learning process (Farrell & Brunton, 2020). Higher levels of cognitive, behavioral, and emotional engagement are significantly related to academic achievement and professional success (Chen et al., 2021; Khan et al., 2023). Numerous research has demonstrated that environmental factors, such as educators, peers, and parents, can effectively influence online academic engagement (Luan et al., 2023; Zhou et al., 2019). Notably, different from traditional online studies, parents were involved more in their children’s learning than teachers and peers during the pandemic, as key actors in education provision have significantly shifted from professionally trained teachers to parents who are either fully or partially confined at home with their children (Najjengo & Buluma, 2021).

As a positive parenting style, parent autonomy support can foster academic engagement (Ryan, Deci, Grolnick, and La Guardia, 2006; Zhou et al., 2019). Parent autonomy support refers to the degree to which parents encourage their children’s volitional functioning and self-endorsement (Marbell-Pierre et al., 2019). Prior research found that parent autonomy support was positively related to academic engagement in online learning settings (Gallman, 2020; Purnomo et al., 2021). However, most existing research has focused on adolescents, with little attention given to undergraduates who also require parental autonomy support for academic success. Particularly within the context of Chinese collectivist culture, parents and children are closely interdependent (Oyserman et al., 2002). The alignment of the relationship between parent autonomy support and academic engagement among Chinese undergraduates with previous findings remains unclear. Furthermore, if such a relationship exists, how does parent autonomy support influence the three sub-dimensions of undergraduates’ academic engagement in an online learning environment?

Importantly, the underlying mechanism of how parent autonomy support influences academic engagement is unknown in online learning. According to Zimmerman’s (1989) self-regulated theory, the process of self-regulated learning is shaped not only by environmental factors but also by reciprocal behavioral events and personal processes. Parent autonomy support is identified as a vital external environmental factor that affects students’ self-regulation (Boti, 2022; Gong & Wang, 2023; Won & Yu, 2018; Wong, 2008), with the potential to enhance academic engagement. Given that, self-regulation may be a powerful candidate for parent autonomy support and academic engagement. Understanding how parent autonomy support influences academic engagement and its mechanism is essential for Chinese undergraduates to adapt online learning environment, maximize their learning potential, and obtain academic success. Therefore, the study would attempt to investigate a) the relationship between parent autonomy support and different dimensions of academic engagement in online learning among Chinese undergraduates; and b) the mediation effect of self-regulation on such relationships.

## Literature review

### Parent autonomy support and academic engagement

Parent autonomy support is a robust predictor of academic engagement (Gao et al., 2021; Khosravi & Mohanan, 2020; Stevens & Borup, 2015; Vasquez et al., 2016). It involves parents’ behaviors including considering children’s perspectives, fostering self-initiation, and encouraging open communication and the expression of opinions (Assor et al., 2002; Grolnick, Ryan, Deci, 1991; Soenens et al., 2007). With parent autonomy support, children are more likely to engage effectively in online learning. Purnomo et al.’ (2021) research investigated 251 fourth and fifth-grade students in Indonesia. Their findings indicated that when parents got involved by guiding and motivating their children, children were more likely to engage cognitively, socially, and emotionally in online mathematics learning. For example, Gao et al. (2021) conducted a study involving 1317 undergraduates and discovered that those who received greater interest and support from their parents exhibited higher levels of engagement in e-learning activities.

Notably, academic engagement is a multifaceted construct. As outlined in the framework proposed by Fredricks et al. (2004), it consists of behavioral, cognitive, and emotional engagement. Hu and Li (2017) further applied Fredricks et al.’s work in online learning. Specifically, cognitive engagement involves the use of learning strategies, like the regulation of mental cognitive effort required for learning and the active application of newly acquired knowledge. Behavioral engagement refers to explicit and observable behaviors during learning activities, such as reading course materials, posing questions, participating in interactive exercises, and completing assignments. Emotional engagement captures the spectrum of students’ emotional responses in learning, including curiosity, boredom, enjoyment, sadness, and worry.

There may be several indirect evidence between parent autonomy support and three sub-dimensions of academic engagement. Regarding cognitive engagement, students perceived more parental involvement tend to proactively use learning strategies and invest greater effort in learning activities (Sahil & Hashim, 2011). This finding was particularly relevant in the context of online learning, where undergraduates are often distracted (Maqableh & Alia, 2021). Through parent autonomy support, undergraduates can be effective in employing self-regulation strategies, setting task-oriented goals, and allocating more mental effort to their learning pursuits. With regard to behavioral engagement in online learning, undergraduates often lack initiative and exhibit worse learning performance. Parent autonomy support can motivate undergraduates to participate in classes and complete assignments with high quality (Reeve, 2009). In terms of emotional engagement, parent autonomy support serves as a protective factor against stress, anxiety, and depression in the online learning environment (Worley & Mucci-Ferris, 2021). With parental support, undergraduates are more likely to have positive emotions and enhance confidence in online learning tasks (Gao et al., 2021; Huang & Zhang, 2022). Notably, although parent autonomy support for undergraduates in online learning is important, previous research has focused on adolescents. For example, Purnomo et al.’s (2021) research found that parent involvement significantly predicted cognitive, social, and emotional engagement in online math learning.

### The mediating role of self-regulation

Self-regulation can significantly predict three sub-dimensions of academic engagement. It refers to the foundational ability to manage dominant impulses and regulate emotions, thoughts, and actions (Baumeister & Vohs, 2007). Students show high levels of self-regulation through various self-control processes, including self-instruction, focused attention, and metacognitive approaches, to optimize problem-solving strategies and enhance cognitive engagement (Li & Lajoie, 2022). Moreover, a high level of self-regulation can stimulate students’ motivation to increase behavioral engagement (Schwinger & Stiensmeier-Pelster, 2012). Additionally, students used self-regulation strategies, like self-talking, to alleviate negative emotions and facilitate emotional engagement. For example, a study conducted by Sun and Rueda (2012), which involved 203 undergraduates participating in online classes, found self-regulation was significantly related to cognitive, behavioral, and emotional engagement.

Parent autonomy support plays a pivotal role in student self-regulation (Gong & Wang, 2023; Won & Yu, 2018). According to self-regulated theory, the interactions among individuals (self), the environment, and behaviors shape self-regulated learning (Zimmerman, 1989). With parent autonomy support, children are more likely to recognize the value of education and foster greater autonomy-based motivation for learning activities (Lerner & Grolnick, 2020). This autonomous learning motivation, in turn, encourages students to regulate and monitor their own learning. Numerous researches consistently demonstrate a positive correlation between parent autonomy support and self-regulation among undergraduates across diverse cultures (Boti, 2022; Gong & Wang, 2023; Won & Yu, 2018; Wong, 2008). For example, Gao et al. (2021) found that self-efficacy mediated the relationship between parental support and e-learning engagement. This finding indicated that students perceived a higher level of parent support can help them to consciously obey the established norms of learning behavior and feel capable of dedicating themselves to learning, thus engaging more in online learning. Similarly, Sahil and Hashim (2011) found parental support can influence cognitive engagement mediated by academic efficacy. These findings provide indirect evidence that parent autonomy support influences three sub-dimensions of academic engagement through self-regulation.

### In the context of Chinese collectivist culture

To investigate the relationship between parent autonomy support and sub-dimensions of academic engagement and the mediating effect of self-regulation on such relationships, it is essential to consider the context of Chinese collectivist culture. During the COVID-19 pandemic, parents need to overcome the challenge that their children must attend online class at home rather than in the university. In this context, the dynamics of parent–child relationships required substantial renegotiation (Pfefferbaum & North, 2020; Rajkumar, 2020). Particularly in Chinese collectivist culture which traditionally emphasizes interdependence (Chao, 1994; Rudy & Grusec, 2006), the coercive nature of these adjustments may result in feelings of depression and dropout among undergraduates. Specifically, excessive parent involvement and controlling behaviors may deprive undergraduates’ need for autonomy and hinder their capability to take on adult responsibility (Love et al., 2020; Reed et al., 2016). Rather than enforcing control, parent autonomy support emerges as a crucial role in assisting undergraduates to cultivate intrinsic motivation, regulate emotions, and manage behaviors (Ma et al., 2022). Importantly, with rapid economic development and cultural transformations in contemporary China, the younger generation gradually recognizes the significance of autonomy support (Nalipay et al., 2020). Chinese parents are increasingly prioritizing their children’s independence, happiness, and emotional well-being (Chen-Bouck et al., 2017). Consequently, further research is necessary to expand current knowledge and explore the relationship between Chinese parent autonomy support and academic engagement among undergraduates.

### The present study

**Fig. 1:**
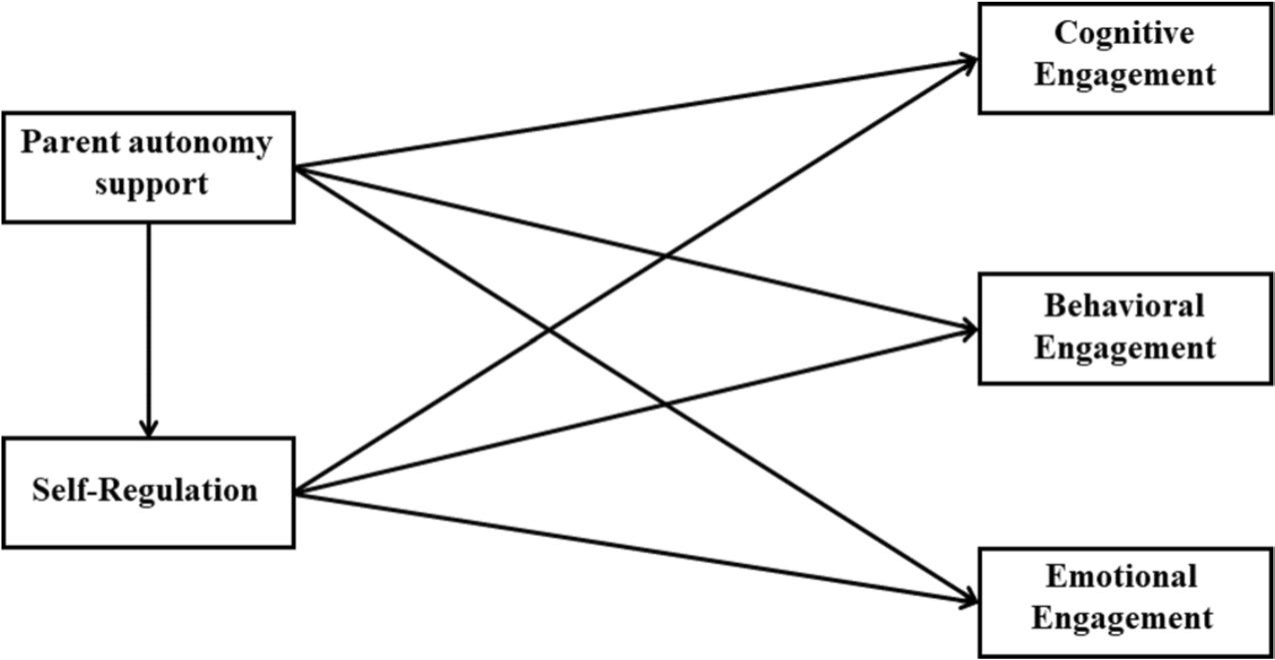
The structural model

During the COVID-19 pandemic, the role of parent support in shaping students’ online learning experiences has attracted unprecedented attention (Novianti & Garzia, 2020; Snaman et al., 2020). Previous research suggested a potential contribution of parent autonomy support to online learning engagement among adolescents, while it is unclear among undergraduates in the specific context. Moreover, the specific dimensions of academic engagement influenced by such support among undergraduates remain unknown. Drawing on Zimmerman’s (1989) self-regulated theory and the perspective of Vasquez et al. (2016), this study posited that self-regulation may serve as a mediating factor in the relationship between parent autonomy support and three sub-dimensions of academic engagement. This study hypothesizes that (1) parent autonomy support predicts the three sub-dimensions of academic engagement among undergraduates and (2) self-regulation mediates the association between parent autonomy support and the three sub-dimensions of academic engagement. The structural model is illustrated in Fig. 1.

## Methods

### Participants and procedure

In the autumn term of 2020, we recruited 1908 undergraduates (*M*_age_ = 19.63, SD = 1.04 years, 1317 females) from 22 universities across the eastern, central, and western regions of China. Participants consisted of 670 freshmen, 691 sophomores and 547 juniors. Ethical approval for this study was obtained from the ethics committee of Shaoxing University. The recruitment process involved distributing questionnaires to students engaged in online English courses through their respective English teachers. Following a comprehensive review of the study statement and obtaining informed consent, undergraduates voluntarily completed the Chinese version of online questionnaires designed to assess parent autonomy support, academic engagement, and self-regulation. Importantly, participants were required to answer all questions before submitting the questionnaire. The entire procedure was conducted on the Wenjuan Xing platform—a platform akin to Amazon Mechanical Turk. It took approximately 15 min. Additionally, respondents filled out the questionnaire anonymously with questions presented in a randomized order.

## Measures

### Measurements

#### Parent autonomy support scale

Parent autonomy support was assessed using a revised Chinese version of the Parent Autonomy Support Scale (Tang et al., 2013), which was originally developed by Steinberg et al. (1992). This scale had 12 items (e.g., “My parents allow me to make decisions for myself”). All items were rated on a 5-point Likert scale, ranging from 1 (not true at all) to 5 (very true). High scores mean high levels of parental autonomy support. This scale has been implemented in Chinese populations and showed good internal consistency (Xu et al., 2019). Composite reliability (CR) was 0.95, exceeding the threshold of 0.70 and indicating strong reliability. In addition, the robust McDonald’s omega was 0.95. Average variance extraction (AVE) was used to test the validity of the questionnaire. AVE was 0.59, exceeding the threshold of and confirming good validity for the scale. Confirmatory factor analysis affirmed the model’s fit, *χ*^*2*^*/*df = 24.40, TLI = 0.91, CFI = 0.93, SRMR = 0.04.

#### Academic engagement scale

Academic engagement was evaluated using the Academic Engagement Scale adapted by Ma et al. (2015) from Lam et al. (2009) for online learning. This scale had 16 items categorized into three sub-dimensions. Cognitive engagement included six items (e.g., “When I study in the online settings, I try to preview new knowledge and related knowledge of other subjects”), behavioral engagement consisted of five items (e.g., “I can concentrate on the course and materials during online learning”), and emotional engagement had five items (e.g., “I feel excited when studying online”). All items were rated on a 5-point Likert scale ranging from 1 (not true at all) to 5 (very true). Previous research supported the scale’s reliability and validity (Li, 2018). CR of cognitive, behavioral, and emotional engagement was 0.94, 0.91, and 0.93, respectively, indicating good reliability. The robust McDonald’s omega values for three sub-dimensions of academic engagement were 0.95, 0.93, and 0.94, respectively. AVE was 0.72 for cognitive engagement, 0.60 for behavioral engagement, and 0.71 for emotional engagement, confirming that the scale possesses strong validity. Confirmatory factor analysis indicated a good model fit, *χ*^*2*^*/*df = 11.43, TLI = 0.96, CFI = 0.96, RMSEA = 0.07, SRMR = 0.03.

#### Self-regulation scale

Self-regulation was assessed using the Metacognitive Self-Regulation (MSR) scale (Tock & Moxley, 2017), which was translated into Chinese by a professional translator. The scale consisted of nine items (e.g., “Even if the learning content is boring, I will exert effort to complete it during online learning”), including two reverse-coded items (e.g., “I often find that I have been reading for class but don’t comprehend what it was all about”). Seven-point Likert was used to rate scores, with scores ranging from 1(“not at all true”) to 7(“very true”). Scores for negative items were reversed before computation. CR achieved a value of 0.94, indicating strong reliability. The McDonald’s omega for this scale was 0.74. AVE was 0.68, suggesting good validity. The model fit indices indicated satisfactory results: *χ*^2^/df = 34.06, TLI = 0.91, CFI = 0.91, RMSEA = 0.13, SRMR = 0.06.

### Data analysis

All data were analyzed using Statistical Package for the Social Sciences (SPSS, version 20.0) and Mplus 7.4 software. Initially, SPSS was utilized to calculate mean values, standard deviations, and Pearson’s correlations. Subsequently, Mplus 7.4 was used to conduct a mediation analysis that examined both direct and indirect effects. Parent autonomy support served as the independent variable, while the three sub-dimensions of academic engagement and self-regulation were designated as dependent and mediating variables, respectively.

## Results

### Descriptive statistics and correlations among main measures

Table 1 presents the correlations and descriptive characteristics of variables. Mean values for parent autonomy support, self-regulation, cognitive, behavioral, and emotional engagement in online learning were 3.80, 4.52, 3.75, 3.71, and 3.81, respectively. Notably, the study found a negative correlation between students’ gender and cognitive engagement, with no significant associations between gender and other variables. Additionally, age was negatively related to parent autonomy support, self-regulation, cognitive engagement, behavioral engagement, and emotional engagement, albeit with small correlation coefficients. Apart from gender and age, all other main variables exhibited significant and positive correlations.

**Table 1:** Correlations and descriptive statistics among main variables

	1	2	3	4	5	6	7	Scale range	*M*	SD	Skewness	Kurtosis
1 Gender	–							–	1.69	0.46	− 0.80	− 1.36
2 Age	0.04	–						–	19.63	1.04	0.47	1.02
3 Parent autonomy support	− 0.01	− 0.09^***^	–					1–5	3.80	0.66	− 0.33	0.78
4 Self-regulation	− 0.03	− 0.12^*^	0.44^***^	–				1–7	4.52	0.62	0.29	0.48
5 Cognitive engagement	− 0.06^*^	− 0.13^***^	0.47^***^	0.65^***^	–			1–5	3.75	0.69	− 0.31	0.65
6 Behavioral engagement	− 0.03	− 0.10^***^	0.49^***^	0.66^***^	0.75^***^	–		1–5	3.71	0.68	− 0.36	0.85
7 Emotional engagement	− 0.04	− 0.09^***^	0.51^***^	0.64^***^	0.78^***^	0.85^***^	–	1–5	3.81	0.67	− 0.32	0.66

^***^p < 0.001, ^*^p < 0.05

### Structural equation model analyses

**Fig. 2:**
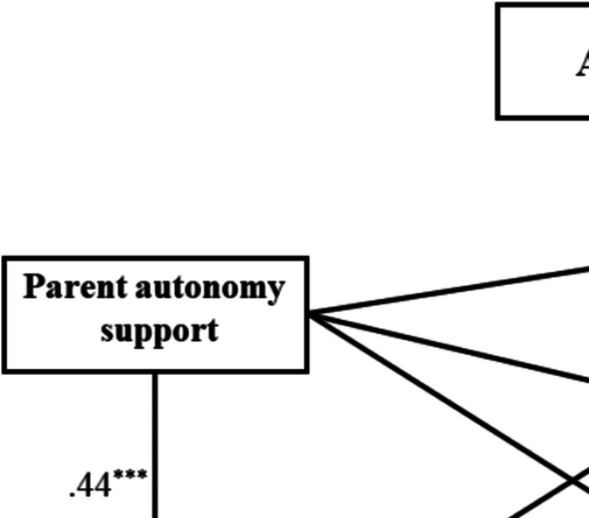
The mediation of self-regulation

To examine self-regulation as a mediator in the relationship between parent autonomy support and three sub-dimensions of academic engagement, a structural equation model was constructed to test the model. The following fit indices were used to evaluate the model fit, as proposed by Hu and Bentler (1999): Chi-Square Test of Model Fit (*χ*^*2*^), Comparative Fit Index (CFI), Tucker–Lewis Index (TLI), and Root Mean Square Error of Approximation (RMSEA) including the 90% Cis of the RMSEA [90% CI]. The constrained model exhibited good fit, *χ*^*2*^ = 6334.48, df = 10, CFI = 1, TLI = 1, RMSEA = 0.000, 90% CI [0.000, 0.000] SRMR = 0.000. In addition, previous research found that gender and age were significantly related to academic engagement (Hu & Kuh, 2002; Lam et al., 2016; Manzano, 2002; Sontam & Gabriel, 2012). Given that, both variables were controlled in the SEM model. After controlling for gender and age, the constrained model had an acceptable fit, *χ*^*2*^ = 17.712, df = 2, *χ*^*2*^*/*df = 8.856, CFI = 0.998, TLI = 0.978, RMSEA = 0.064, 90% CI [0.039, 0.093], SRMR = 0.020 (Fig. 2).

### Analysis of the direct effect

To test the standardized total (direct and indirect) effects, we implemented a bootstrapping procedure with 2000 iterations taken from our sample. If the estimates of 95% confidence interval excluded zero, indicating the mediation effect was significant. The results in Table 2 show the standardized direct effect among all variations. The results revealed that parent autonomy support had positive and significant effects on cognitive, behavioral, and emotional engagement (*ß* cog = 0.232, *p* < 0.001; *ß* beh = 0.250, *p* < 0.001; *ß* emo = 0.284, *p* < 0.001).

**Table 2:** The direct effect of parent autonomy support on cognitive, behavioral, and emotional engagement

Dependent variable	Independent variable	coefficients	se	*t*	*p*	95%CI
Self-regulation (M)	PAS (X)	0.437	0.022	19.579	< 0.001	[0.393, 0.478]
Cognitive engagement (Y1)	PAS (X)	0.232	0.022	10.293	< 0.001	[0.188, 0.277]
Behavioral engagement (Y2)	PAS (X)	0.250	0.022	11.389	< 0.001	[0.208, 0.294]
Emotional engagement (Y3)	PAS (X)	0.284	0.022	12.920	< 0.001	[0.241, 0.327]

*PAS* parent autonomy support

### Analysis of indirect effects

The current study examined the mediating effect of self-regulation between parent autonomy support and three sub-dimensions of academic engagement in online learning. The results presented in Table 3 showed the significant indirect effects. Specifically, the indirect effect of parent autonomy support on cognitive engagement via self-regulation was significant (*ß* cog = 0.236, *p* < 0.001), with the mediation effect accounting for 50% of the total effect. Additionally, the indirect effect of parent autonomy support on behavioral engagement via self-regulation was significant (*ß* beh = 0.241, *p* < 0.001). The mediation effect accounted for 49% of the total effect. Finally, the indirect effect of parent autonomy support on emotional engagement via self-regulation was significant (*ß* emo = 0.224, *p* < 0.001). The mediation effect accounted for 44% of the total effect.

**Table 3:** The indirect effect of parent autonomy support on cognitive, behavioral, and emotional engagement

Paths	Coefficients	se	*t*	*p*	95%CI	Proportions of total indirect effect
PAS → Self-regulation → CE	0.236	0.015	15.693	< 0.001	[0.206, 0.266]	50%
PAS → Self-regulation → BE	0.241	0.015	16.073	< 0.001	[0.212, 0.271]	49%
PAS → Self-regulation → EE	0.224	0.014	15.612	< 0.001	[0.198, 0.254]	44%

*PAS* parent autonomy support, *CE* cognitive engagement, *BE* behavior engagement, *EE* emotional engagement

## Discussion

The study examined the effects of parent autonomy support on three sub-dimensions of academic engagement and the mediating effects of self-regulation in online learning. The result remained significant even after controlling for grade and gender. The current study first explored that parent autonomy support was positively related to three sub-dimensions of academic engagement among undergraduates in online learning.

### Parent autonomy support and three sub-dimensions of academic engagement

Parent autonomy support was positively related to cognitive, behavioral, and emotional engagement among undergraduates in online settings, supporting Hypothesis 1. This finding was consistent with Gao et al. (2021), revealing that Chinese undergraduates who perceived higher parent support demonstrated enhanced e-learning engagement. Furthermore, our study systematically investigated the effect of parent autonomy support on cognitive (*ß* = 0.232), behavioral (*ß* = 0.250), and emotional (*ß* = 0.284) engagement, which the medium effect size showed the strong relationships among those variables even after controlling for grade and gender. In collective culture, traditional Chinese parents are heavily influenced by Confucian culture and tend to adopt a controlled parenting style (Lin et al., 2020). However, with the rapid development of the economy and the popularization of the family concept, parents of the younger generation pay more attention to the comprehensive development and psychological well-being of their children (Yu et al., 2018). Instead of controlling their children, parents of the younger generation begin to adopt the new parenting style that grants their children more autonomy to navigate their own paths (Way et al., 2013). First, our study found that parent autonomy support has the largest impact on emotional engagement. A possible explanation for this finding is that undergraduates’ emotional engagement is most readily perceived by their parents. During the pandemic, children experience feelings of isolation, despondency, and anxiety. When perceiving children’s negative emotions, Chinese parents provide timely psychological counseling, like listening and empathic understanding. With parent autonomy support, undergraduates show resilience in overcoming challenges and receive emotional support from parents, which facilitates emotional engagement in online learning. Second, parent autonomy support influenced online behavioral engagement with a medium effect size of 0.25. Behavior engagement is relatively apparent and easily observable by parents. When parents notice a decline in their child’s behavioral engagement, they can provide feedback after class to help undergraduates adjust in subsequent classes. In this way, undergraduates benefit from autonomy support by actively motivating themselves to participate in online classes, thereby enhancing behavioral engagement. Third, we found that parent autonomy support had the least effect on cognitive engagement, but the effect size remained above 0.20. This may be attributed to the fact that parents cannot directly enhance their children’s attention during class without potentially disrupting it. Instead, parents provide long-term autonomy support and encouragement, which enables undergraduates to allocate their mental effort judiciously and fosters cognitive engagement. In summary, these results underscore the pivotal role of parent autonomy support in influencing cognitive, behavioral, and emotional engagement among undergraduates in online learning.

### The mediating role of self-regulation

This study found the mediating effect of self-regulation between parent autonomy support and three sub-dimensions of academic engagement, supporting Hypothesis 2. The findings of the mediation effect were consistent with the self-regulated theory (Zimmerman, 1989). In online learning, undergraduates who received parent autonomy support demonstrated higher levels of self-regulation. As core supervisors in online learning, parents offer autonomy support that helps children recognize the intrinsic value of learning and actively regulate themselves (Lerner & Grolnick, 2020). Previous research indicated that parent autonomy support promoted adolescent’s development of self-regulation (Liew et al., 2014). Heightened parent autonomy support offers more opportunities for children to make better decisions (Won & Yu, 2018). Particularly during periods of stress in online learning, parent autonomy support serves as an encouragement for students to make informed decisions that are adapted to the current context (Bashir, Malik, and Atta, 2021). Additionally, our results indicated a positive correlation between self-regulation and three sub-dimensions of academic engagement. This was consistent with the study conducted by Sun and Rueda (2012), yet it contrasted with the research presented by Pellas (2014). Sun and Rueda (2012) found a positive connection between metacognitive self-regulation and emotional and cognitive engagement, whereas Pellas (2014) identified a negative association with behavioral engagement in online learning. This discrepancy might be attributed to variations in levels of students’ self-regulation, where lower levels correspond to increased academic challenges (Park & Kim, 2022). Enhanced self-regulation enables undergraduates to exert greater mental effort, improve concentration in class, listen attentively to lectures, and manage negative emotions during online learning. Notably, this study reveals the novel insight that parent autonomy support influences the three sub-dimensions of academic engagement through the mechanism of self-regulation in online learning.

### Implication

Theoretically, this study verified the self-regulated theory that environmental factors (i.e., parent autonomy support) can predict self-regulation, further influencing behavioral performance (i.e., cognitive, behavioral, and emotional engagement) in online learning. The study further supported that self-regulated theory was also applied to the online environment among Chinese undergraduates. In addition, this study also provides a theoretical basis for parents who are self-employed and undergraduates who study online at home. Autonomy support from parents can encourage students to be more engaged in their online learning activities.

Moreover, these findings have practical implications for parents in creating an autonomous supportive learning environment that fosters students’ self-regulation to increase their participation in online learning. Based on the results, the current study has significant implications for both parents and higher education institutions.

First, these findings suggest that parents should provide autonomy support for their college-aged children’s online academic engagement. It is advisable for Chinese parents to shift away from negative strategies, such as psychological control and excessive intervention. They should focus on recognizing their children’s genuine learning needs and provide appropriate guidance along with feasible strategies, such as establishing an independent and quiet learning environment.

Second, it is essential for Chinese parents to recognize the significance of fostering their children’s self-regulation. Specifically, they need to establish the awareness that children are the masters of learning and are responsible for their own learning. Parents can minimize controlling behaviors during their child’s educational activities and instead implement supportive strategies that honor the child’s autonomy when faced with less enjoyable tasks (Joussemet et al., 2008).

Third, there are several recommendations about higher education for undergraduates. The Ministry of Education could set up an online platform and provide relevant family education courses for parents and undergraduates to face the challenge of online learning. In this way, parents can systematically learn how to support their children properly and undergraduates also can acquire knowledge to obtain autonomy support from parents, which can create a healthy and harmonious environment for academic engagement in online learning. Moreover, the university counseling center should focus on undergraduates with low parental autonomy support. Providing increased encouragement and affirmation can help those undergraduates recognize the value of their efforts and enhance their confidence in learning. Furthermore, it is essential to guide students who benefit from high parental autonomy support. By effectively using external assistance, they are more likely to succeed in the learning process.

## Limitations and future research

Although this study first found that parent autonomy support influenced academic engagement through self-regulation in online learning, there are several limitations. First, the cross-sectional design prevents drawing causal conclusions from the results. Further research is needed to clarify the causal relationships between variables. Second, this study did not distinguish types of self-regulation, despite its multifaceted nature, which includes emotional and cognitive dimensions (Gong & Wang, 2023; Liebermann et al., 2007). Future studies could be conducted to elucidate these different types of self-regulation. Third, although self-regulation is reliable according to the framework of Field (2005), the model fit for the self-regulation scale is questionable due to the high RMSEA value (over the acceptable value of 0.08). Therefore, it is essential that the self-regulation scale undergoes further modification within the context of China.

## Conclusion

Parent autonomy support is a promising factor to promote high levels of academic engagement in online learning. However, most existing research has paid less attention to undergraduates, particularly in Chinese collective culture. Moreover, few studies have systematically investigated the relationship between parent autonomy support and academic engagement in an online environment, given the various dimensions of academic engagement. Additionally, the underlying mechanisms linking parent autonomy support to academic engagement remain unclear. To address these research gaps, the present study collected cross-sectional data to examine these relationships and the mediating role of self-regulation. This study found that exposure to a higher level of parent autonomy support was positively related to three sub-dimensions of academic engagement among undergraduates in online learning. These findings showed that parents should autonomously support their children to promote their cognitive, behavioral, and emotional engagement. Moreover, self-regulation mediated the relationship between parent autonomy support and academic engagement. The mediating pathway provides a comprehensive understanding of how parent autonomy support influences three sub-dimensions of academic engagement. The findings recommend that parents should adopt an autonomy-supportive parenting style to foster their children’s self-regulation, which can further enhance their online academic engagement.

## Supplementary Information

Supplementary Material 1.

## Data Availability

The datasets used and/or analyzed during the current study are available from the corresponding author upon reasonable request.

## Abbreviations

PAS: Parent autonomy support
SR: Self-regulation
CE: Cognitive engagement
BE: Behavior engagement
EE: Emotional engagement
