# Supplementary material for: Parent autonomy support and undergraduates’ academic engagement in online learning: the mediate role of self-regulation
Source: Psicol Reflex Crit. 2024 Nov 18;37:45. doi: 10.1186/s41155-024-00330-1 (PMC11573943; doi:10.1186/s41155-024-00330-1)
Supplement: Supplementary file 1 — Supplementary Material 1. [file 41155_2024_330_MOESM1_ESM.docx]

**Supplementary material**

We calculate discriminant validity by comparing each factor's AVE to the square of correlations between factors. Moreover, we present the factor structure of each scale with the standardized regression weights in the, as well as correlations and covariances matrix of each scale's items.

**Self-regulation**

**Composite reliability (CR)** of self-regulation was .94.

**Average variance extracted (AVE)** of self-regulation was .68.

**Standardized Regression Weights**

|  | Estimate |
| --- | --- |
| Sr1 <--- self-regulation | .763 |
| Sr2< --- self-regulation | .255 |
| Sr3< --- self-regulation | .777 |
| Sr4< --- self-regulation | .835 |
| Sr5< --- self-regulation | .802 |
| Sr6< --- self-regulation | -.270 |
| Sr7< --- self-regulation | -.213 |
| Sr8< --- self-regulation | .556 |
| Sr9< --- self-regulation | .445 |

**Covariances**

|  | Estimate | S.E. | C.R. | P |
| --- | --- | --- | --- | --- |
| e6 <--> e7 | .933 | .047 | 19.933 | *** |
| e2 <--> e6 | -.978 | .051 | -19.339 | *** |
| e2 <--> e7 | -.918 | .050 | -18.489 | *** |
| e7 <--> e8 | .287 | .028 | -10.110 | *** |

**Correlations**

|  | Estimate |
| --- | --- |
| e6 <--> e7 | .506 |
| e2 <--> e6 | -.501 |
| e2 <--> e7 | -.461 |
| e7 <--> e8 | -.203 |

**Parent autonomy support**

**Composite reliability (CR)** of parent autonomy support was .95.

**Average variance extracted (AVE)** of parent autonomy support was .59.

**Standardized Regression Weights**

|  | Estimate |
| --- | --- |
| ps12 <--- pas | .764 |
| ps11 <--- pas | .788 |
| ps10 <--- pas | .852 |
| ps9 <--- pas | .848 |
| ps8 <--- pas | .733 |
| ps7 <--- pas | .713 |
| ps6 <--- pas | .534 |
| ps5 <--- pas | .82 |
| ps4 <--- pas | .832 |
| ps3 <--- pas | .696 |
| ps2 <--- pas | .825 |
| ps1 <--- pas | .762 |

**Academic engagement**

**Composite reliability (CR)** of academic engagement was .94.

**Average variance extracted (AVE)** of academic engagement was .68.

**Standardized Regression Weights**

|  | Estimate |
| --- | --- |
| ae1 <--- academic engagement | 0.716 |
| ae2 <--- academic engagement | 0.846 |
| ae3 <--- academic engagement | 0.767 |
| ae4 <--- academic engagement | 0.865 |
| ae5 <--- academic engagement | 0.871 |
| ae6 <--- academic engagement | 0.861 |
| ae7 <--- academic engagement | 0.881 |
| ae8 <--- academic engagement | 0.845 |
| ae9 <--- academic engagement | 0.775 |
| ae10 <--- academic engagement | 0.849 |
| ae11 <--- academic engagement | 0.818 |
| ae12 <--- academic engagement | 0.861 |
| ae13 <--- academic engagement | 0.875 |
| ae14 <--- academic engagement | 0.872 |
| ae15 <--- academic engagement | 0.818 |
| ae16 <--- academic engagement | 0.827 |

**Covariances**

|  | Estimate | S.E. | C.R. | P |
| --- | --- | --- | --- | --- |
| BE <--> EE | .397 | .017 | 23.131 | *** |
| EE <--> CE | .363 | .015 | 23.567 | *** |
| BE <--> CE | .323 | .015 | 21.163 | *** |

**Correlations**

|  | Estimate |
| --- | --- |
| BE <--> EE | .933 |
| EE <--> CE | .827 |
| BE <--> CE | .787 |
